# Functional organisation of *Escherichia coli* transcriptional regulatory network

**DOI:** 10.1016/j.jmb.2008.05.054

**Published:** 2008-08-01

**Authors:** Agustino Martínez-Antonio, Sarath Chandra Janga, Denis Thieffry

**Affiliations:** 1 Departamento de Ingeniería Genética, Centro de Investigación y de Estudios Avanzados, Instituto Politécnico Nacional, Campus Guanajuato, Irapuato 36500, México; 2 MRC Laboratory of Molecular Biology, Hills Road, Cambridge CB2 0QH, UK; 3 TAGC U928, INSERM and Université de La Méditerranée, Campus Scientifique de Luminy, Case 928, 13288 Marseille, France; 4 CONTRAINTES Project, INRIA Paris-Rocquencourt Center, domaine de Voluceau, BP 105, 78153 Le Chesnay, France

**Keywords:** TF, transcription factor, CRP, cAMP receptor protein, cAMP, cyclic adenosine monophosphate, FFL, feed-forward loop, HNS, histone-like protein, FNR, fumarate and nitrate regulatory protein, IHF, integration host factor, transcriptional regulation, network organisation, multiple phenotypes, homeostasis

## Abstract

Taking advantage of available functional data associated with 115 transcription and 7 sigma factors, we have performed a structural analysis of the regulatory network of *Escherichia coli*. While the mode of regulatory interaction between transcription factors (TFs) is predominantly positive, TFs are frequently negatively autoregulated. Furthermore, feedback loops, regulatory motifs and regulatory pathways are unevenly distributed in this network. Short pathways, multiple feed-forward loops and negative autoregulatory interactions are particularly predominant in the subnetwork controlling metabolic functions such as the use of alternative carbon sources. In contrast, long hierarchical cascades and positive autoregulatory loops are overrepresented in the subnetworks controlling developmental processes for biofilm and chemotaxis. We propose that these long transcriptional cascades coupled with regulatory switches (positive loops) for external sensing enable the coexistence of multiple bacterial phenotypes. In contrast, short regulatory pathways and negative autoregulatory loops enable an efficient homeostatic control of crucial metabolites despite external variations. TFs at the core of the network coordinate the most basic endogenous processes by passing information onto multi-element circuits. Transcriptional expression data support broader and higher transcription of global TFs compared to specific ones. Global regulators are also more broadly conserved than specific regulators in bacteria, pointing to varying functional constraints.

## Introduction

In bacteria, coupling of gene expression with external conditions is achieved through two molecular functions: (i) binding of transcription factors (TFs) at specific sites in the genome and (ii) recognition of a relevant effector signal or metabolite.^1,2^ Typically, these functions are performed by different domains of a single polypeptide, but there are also cases where two interacting proteins are responsible for these functions, as in two-component systems.^3^

At the phenotypic level, there are evidences for the coexistence of multiple phenotypes in bacterial cultures, e.g., of cells with different morphological and physiological abilities such as motility, biofilm formation, drug resistance, etc.^4,5^ In particular, biofilm formation and chemotaxis are considered as multistage developmental processes, and in mature biofilms, a mixture of bacterial population from different developmental stages were found to coexist.^6,7^

Here we take advantage of the wealth of experimental data on transcriptional regulation for the best-characterized bacterium, *Escherichia coli*, to analyse the structure of the transcriptional network in light of different functional constraints.

## Results and Discussion

### Topology of *E. coli* cross-regulatory transcriptional network

**Fig. 1:**
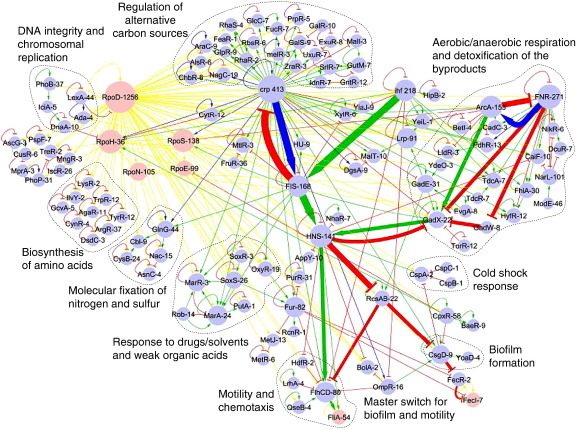
Core transcriptional regulatory network of *E. coli*. Blue and pink nodes represent genes encoding for TFs and sigma factors, respectively; each node label is accompanied with its connectivity showing the number of regulatory targets. Edges represent cross-regulatory interactions (green for activation, red for repression, blue for dual interactions and yellow for sigma transcription), whereas loops represent transcriptional autoregulations. Specific subnetworks, such as the one associated with the regulation of carbon sources, are delineated with dashed lines to distinguish different regulatory modules. This figure was generated using Cytoscape.^14^

Available experimental data point to more than 3000 regulatory interactions between TFs and their regulated genes in *E. coli*. This information is integrated and documented in a specialised database called RegulonDB.^8^ Global analyses of this huge network have already been published, emphasising a hierarchical organisation and statistically overrepresented regulatory motifs.^9–11^ Here, our aim is to analyse the flow of regulatory information within the network of transcriptional interactions among TFs and sigmas (*E. coli* transcriptional cross-regulatory network). This network encompasses 115 TFs and 7 sigma factors, i.e., around one-third of the total predicted TF proteins in this bacterium (Fig. 1).^12,13^ On average, every TF is connected to two other TFs (i.e., more technically, the mean degree of the regulatory graph is 2.74). However, the connectivity distribution of TFs is not uniform, with a small fraction of global TFs with high out-degrees dominating the network.^15^ Seven global regulators were defined previously based on a collection of criteria:^15^ (i) number of regulated genes; (ii) number of regulated genes encoding for TFs; (iii) propensity of cooperative regulation of targets with the aid of other TFs; (iv) ability to directly affect the expression of a variety of promoters that use different sigma factors; (v) belonging to evolutionary families with few paralogs; and (vi) heterogeneity of the functional classes of the regulated genes.

In order to better visualise the informational flow through the network, the following graphical conventions have been used in Fig. 1 (see also legend): (i) the size of the nodes representing TFs is proportional to the number of genes they regulate [e.g., cAMP receptor protein (CRP) regulates 413 genes and is represented by the second biggest node, after the housekeeping sigma factor rpoD]; (ii) arrows and colours refer to the direction and sign of the regulatory interaction; (iii) arrow thickness is proportional to the impact of the interaction, computed as the number of genes thereby (in)directly regulated.

The majority of the TFs in this network are autoregulated (∼ 70%), of which about two-thirds account for negative loops (see Table 1). This finding is consistent with the results of an analysis performed with a much smaller number of TFs about 10 years ago.^17^ This predominance of negative autoregulatory loops contrasts with the predominance of positive arcs between different TFs (about 54%, see Table 1). The dominance of positive regulatory interactions in the regulatory network of *E. coli* is not limited to those among TFs, as when we compute the regulation of all the target genes (3017 arcs) we found that about 54% (1630) are positively regulated, 40% (1206) are repressed, while about 6% (171) are dual regulated. This is especially interesting because a majority of the TFs in bacteria have been reported to act as repressors.^12,18,19^ The conventions used in Fig. 1 clearly display the hierarchical organisation of the network, with master regulators such as CRP, fumarate and nitrate regulatory protein (FNR) or integration host factor (IHF) each (in)directly regulating a large number of other TFs. Furthermore, the layout emphasises important variations regarding the length of the transcriptional cascades.

**Fig. 2:**
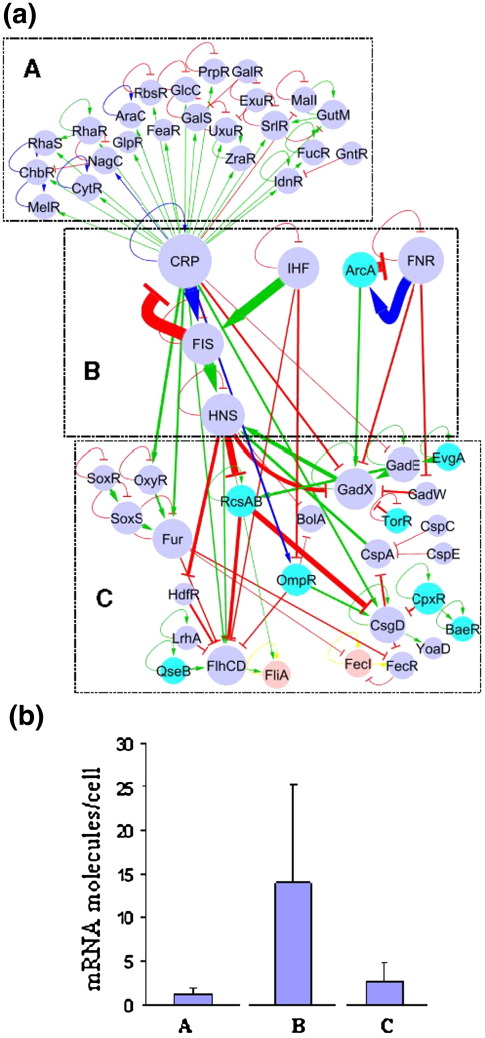
Functional organisation of *E. coli* core transcriptional network. (a) selection of carbon source (group A), global regulation (group B), and regulation of developmental processes (biofilm and chemotaxis, group C). (b) Average mRNA levels per cell for each TF group defined in (a), together with standard deviations. Levels of mRNA were recovered for 13 TFs (56%) of members of group A, all TFs of group B, and 13 TFs (54%) of group C. Nodes in light blue represent members of two-component systems.

Although functional annotations on TFs are still limited, it is possible to classify the cross-regulating TFs into broad categories according to the physiological functions of the target structural genes: carbohydrate initial catabolism, respiration, biofilm formation and chemotaxis, etc. As shown in Fig. 1, these broad classes correspond to different local network topologies. Due to their contrasting topologies, in what follows, we will focus our discussion on short regulatory cascades observed in the case of carbohydrate catabolism as opposed to long regulatory cascades seen in the case of biofilm and chemotaxis pathways (Fig. 2a). CRP resides at the top of both subnetworks. CRP is the only global TF acting hierarchically over local TFs for the usage of carbohydrates, whereas CRP's activity is comparable to the activity of other global regulators in the rest of the network. Note that the concentration of its effector metabolite, cyclic adenosine monophosphate (cAMP), is at par with that of adenosine triphosphate (ATP), which acts as the energetic currency of the cell.^20^ This suggests that CRP not only regulates the use of these substrates for producing ATP, but also senses the energetic status of the cell to decide the execution of other cellular programs.

This study aims at understanding the network structure in relation to physiological roles played by the different modules, focusing on differences in the topologies of the subnetworks controlling metabolism *versus* motility and chemotaxis (cf. the following sections).

**Fig. 3:**
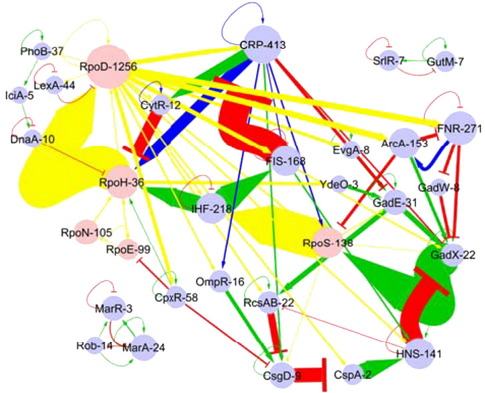
Multi-element regulatory circuits found in the cross-regulatory network of *E. coli*. Extent of informational flux between two nodes is denoted by the thickness of the edges.

However, other subnetworks are also worth mentioning. In particular, all nine TFs controlling the expression of genes for amino acid biosynthesis seem to be expressed constitutively by sigma 70. Each TF regulates the transcription of the required genes for producing different amino acids. At high concentrations of the amino acids, allosteric modifications of TFs follow binding to their respective amino acids, resulting in TF autorepression as well as to the repression of the corresponding biosynthetic genes. Interestingly, the logic behind negative autoregulation in this case is different from that of the catabolism of carbohydrates. While in the latter case TFs are autorepressed until the substrate is available, in the case of amino acids, TFs are autorepressed only in the presence of an excess of the synthesized final product. Another interesting subnetwork is that for alleviating the stresses by drugs, solvents and weak organic acids. The regulatory logic in this complex subnetwork is peculiar, as their components form multi-element circuits (see Fig. 3) and their inputs are directed by Rob and SoxR, two small proteins constitutively expressed but with very short half-lives (1–2 min). Their stability/degradation depends on the presence/absence of their effector signals.^21,22,23^

### Multiple parallel feed-forward loops regulate the use of different carbon sources

Cellular feeding, which includes the uptake of carbon and energy sources and their metabolism, can be considered as one of the main physiological processes in bacterial systems. The regulation of these processes directly affects cellular fitness. The selection of carbon sources is regulated by CRP and about 20 more specific TFs (Fig. 2a). The hierarchical organisation of the corresponding subnetwork is characterized by a short average path length (cf. Table 1). Regulatory interactions between CRP and the specific TFs result in the occurrence of multiple feed-forward loops (FFLs) for the use of alternative sugar sources. FFL is a network motif recurrently found in transcriptional networks and is defined as a three-gene pattern composed of two input TFs, one of which regulates the other, both jointly regulating a target gene.^9,24^ Based on the mode of regulation of each TF, this motif is subdivided into eight different subtypes.^24^ Coherent FFL type 1 corresponds to all the regulatory interactions in the motif being positive; in incoherent type 1 FFL, the first TF regulates positively both the targets, although the second TF represses the expression of the target gene thereby reversing the final effect. The majority of the FFLs present in the subnetwork for carbon catabolism belong to coherent and incoherent type 1 groups,^24^ with both TFs working together, as a result of a persistent signal affecting the global TF (in this case, cAMP) and the presence of a signal affecting a TF corresponding to a sugar alternative to glucose.^24–26^ This motif structure enables the filtering of short pulses of the signal affecting the global TF (cAMP) in case of transient glucose deprivation. Consequently, the target structural genes are activated only in the persistent absence of glucose and in the presence of an alternative carbon source.

The phosphotransferase system typically transports and phosphorylates certain sugars, including glucose, a preferred carbon source for *E. coli*, and this condition ultimately results in low levels of cAMP. Consequently, CRP does not activate the transcription of the genes responsible for the degradation of alternative sugars. Note that most structural genes involved in the transport and initial catabolism of alternative carbon sources are encoded in operons, each specifically repressed in the absence of the inducing sugar.

When glucose is lacking, cAMP level increases and CRP can activate the transcription of genes responsible for degrading alternative carbon sources.^27^ Simultaneously, sugars (or a processed variant thereof) present in the cell bind their specific TF; allosteric interactions then result in TF unbinding from DNA, alleviating the repression and permitting the transcription of the corresponding target genes. This organisation involving multiple parallel FFLs coupled to phosphotransferase activity appears optimal for enabling rapid transcriptional responses to sudden lack of glucose in the presence of alternative carbon sources in the milieu.^28,29^

### Long hierarchical cascades regulate developmental processes

Biofilm formation and bacterial mobility can be seen as the outcome of specialised cell differentiation pathways. Biofilm formation involves subsequent cellular changes at the morphological and physiological levels resulting in bacterial populations with multiple phenotypes.^4,30,31^ Furthermore, bacteria living in biofilm communities are present in different developmental stages (at least four defined stages) as has been observed in *Cryptoccocus*, *Pseudomonas*, *Staphylococcus*, *Xanthomonas*, etc.^32–37^

The part of *E. coli* transcriptional cross-regulatory network involved in the control of biofilm formation and motility exhibits a relatively complex topology with several long cascades from CRP, IHF and FNR to downstream specialised TFs (see Table 1 and Fig. 2a). Several of these cascades converge on the master regulators for motility and biofilm formation (FhlCD and CsgD, respectively).

Furthermore, a relatively high proportion of the downstream specialised TFs autoactivate themselves (see below), a feature that is rare at the level of the whole transcriptional network. In addition, we could identify 105 multi-element circuits in the transcriptional regulatory network involving 2 to 14 different elements (including transcription and sigma factors, see Fig. 3 and supplementary material for the complete list of circuits). Interestingly, the information fluxes inside these circuits follow a frequent route in the network with the order CsgD > Cspa > HNS > GadX > RpoS > IHF. From IHF the regulatory flow diverge in two main directions: IHF > RpoH > RpoD and IHF > FIS > CRP. In line with this observation, CRP and RpoD are the major distributors, whereas CsgD and GadX are the main collectors of information. These multi-element circuits inside the cross-regulatory network are novel observations. The functional relevance of these regulatory structures remains to be assessed experimentally. Tentatively, these circuits may implement a feedback between the presence of different stresses and the basic machinery for replication and growth.

CsgD, the master regulator for biofilm formation, is directly involved in 28 of these long circuits, suggesting a particular tight coupling of CsgD activity with the intracellular status. In contrast, FlhCD, the master compound regulator for motility and chemotaxis, is known to be regulated by nine other TFs but has not yet been reported to regulate any other TF.

Note that the motility module has its own sigma factor, FliA, regulated by FlhCD. FliA is required for the transcription of the genes required for the last part of flagella development and for chemotaxis machinery.^38,39^ In contrast, the genes for biofilm development are transcribed by the housekeeping sigma 70 and RpoS, the sigma factor expressed in response to general stress.^40^

The execution of such long regulatory cascades requires time. Indeed, complete flagella assembly may take a generation time or longer.^41–43^ The occurrence of positively autoregulated TFs at several intermediate steps enables informed decisions about the cellular/environmental condition. In some conditions, cellular duplication might be faster than the conclusion of a long regulatory cascade. This implies that bacterial populations likely consist of mixtures of bacteria with transcriptional programs at different levels in long regulatory cascades.

### Multiple conditions regulate the first steps of developmental processes in *E. coli*

Both biofilm and motility are regulated by multiple, long regulatory cascades (Fig. 2a). This organisation is congruent with the observation that these developmental processes are regulated by multiple environmental conditions, ranging from nutritional deprivation to environmental stresses.^6,7,43^ In *E. coli*, several global TFs sit at the top in these regulatory cascades, namely, CRP, IHF and FNR, all sensing endogenous signals.^2^ Additional regulatory inputs are provided by more specialised TFs such as ArcA (aerobic respiration regulatory protein), EvgA (environmentally responsive activator) and TorR (trimethylamine N-oxide reductase regulator) involved in exogenous sensing. Remarkably, downstream players are enriched in two-component system partners: RcsAB (capsule biosynthesis regulator), OmpR (outer membrane protein regulator), CpxR (regulator of cell envelope proteins folding and degradation) and QseB (quorum sensing regulator) (see the next section). Consequently, the first developmental steps likely occur in most of the cellular population in response to nutritional (CRP) or respiration (FNR-ArcA) stresses, yet depending on nucleoid structure status (sensed through IHF), while the final steps critically depend on exogenous conditions, as observed in other bacteria.^7,44^ For instance, in *Pseudomonas aeruginosa*, the initial steps for biofilm development (motility and reversible attachment to solid surfaces) are independent of quorum sensing, whereas the final steps (irreversible attachment and biofilm maturation) are strongly dependent on quorum-sensing regulation, an exogenous condition.^45^ Both processes (motility and biofilm) must be tightly interconnected, since motility is important for the concerted movement of groups of bacteria over solid surfaces, followed by cell membrane modification, excretion of polysaccharides, causing them to be less motile and smaller in mature biofilms.^46–48^ Interestingly, in *E. coli*, the activation of nucleoid-associated proteins such as histone-like protein (HNS) occurs relatively early along both biofilm and chemotaxis developmental pathways. The expression of these nucleoid-associated proteins is known to be growth-phase dependent (HNS is mostly expressed in the exponential phase, whereas IHF and factor for inversion stimulation (FIS) are expressed in arrested growing cells and during the early exponential phase, respectively), pointing to an influence of nucleoid structure and specific bacterial growth phases on the first steps of these developmental processes.^42,49,50^

### External input information defines the last steps of the developmental processes

Downstream of biofilm and chemotaxis developmental cascades, transcriptional autoactivations and cross-inhibitions become more frequent (Figs. 1 and 2a). This part of the network comprises several two-component systems, enabling the sensing of external information. In particular, OmpR is responsible for switching the phenotype between biofilm and motility (activating the first and repressing the latter) depending on external stresses. During stressing conditions as well as in mature biofilms, cells tend to be smaller compared to rapidly growing cells. This phenotypic transition depends on the expression of BolA (cell morphogenetic regulator), which is transcribed by RpoS and repressed by OmpR.^51^

The activation of the master gene for motility, FlhCD, is further controlled by the quorum-sensing detector QseB, while the activity of the master regulator for biolfilm formation, CsgD, is repressed in the presence of extracellular stressing conditions, detected by CpxR. In addition, the BaeSR system controls the expression of export complexes, conferring multidrug resistance phenotypes,^52^ while RcsB mediates the glutamate-dependent acid resistance.^53,54^ QseB, CpxR, BaeR, OmpR and RcsAB are all members of two-component systems that are modified by protein–protein phosphorylation, enabling quick response in comparison to *de novo* protein production along transcriptional cascades. Strikingly, almost all of these two-component TFs (except OmpR) autoactivate their expression, an observation fitting the contention that positive feedback circuits are necessarily found at the core of all differentiation switches. Indeed, such TF autoactivations likely enable persistent TF expression in two stable states: “on” or “off”. Thus, they constitute a potentially robust machinery for all-or-nothing output response depending on transient exogenous signals.^55–62^

### Expression of master regulators

In the preceding sections, we have emphasised the hierarchical organisation as well as important local structural variations in *E. coli* transcriptional network. In the subsequent section, we consider the expression of a TF as a function of its position in the regulatory hierarchy. Although it is difficult to address this at the protein level because of the current relative scarcity of proteomic data, this question can be readily addressed at the level of transcription. Therefore, we have analysed the levels of mRNA reported by two independent experiments on *E. coli* cells grown on minimal medium + glucose.^63–65^ In summary, the mean mRNA levels of global TFs such as CRP, FNR, ArcA and IHF are significantly higher than those of more specialised regulators (Fig. 2b). This result supports the contention that the master regulators are continuously required at high levels to bind to numerous DNA sites across the genome, whereas downstream, specialised TFs can be expressed or not depending on the environmental conditions. Indeed, in conditions favourable to planktonic growth (as in the condition for mRNA quantification mentioned above), TFs controlling the final stages of biofilm/motility development are lowly expressed. Similarly, the TFs controlling specific sugar catabolism regulons show much lower mean concentration compared to CRP, suggesting that these TFs are sporadically required, depending on the metabolic state of the cell. A similar tendency has been recently observed by measuring the fluorescence of 15 different TF–green fluorescent protein gene fusions.^66^

### Conservation of the transcriptional cross-regulatory network in bacteria

**Fig. 4:**
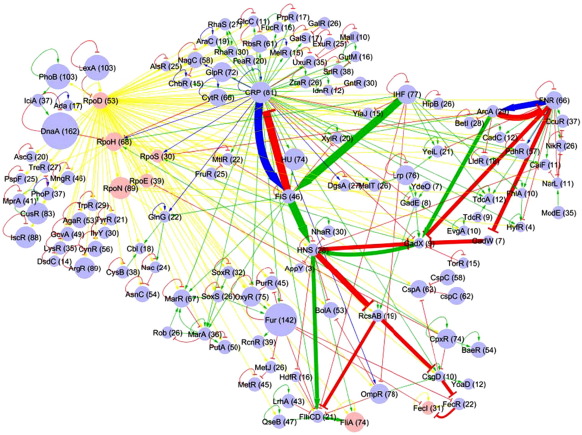
Conservation of *E. coli* sigma and transcription factors across 216 non-redundant bacterial genomes. Node sizes are proportional to the corresponding conservation interval and to the number of genomes in which an ortholog was found (shown in parentheses).

To what extent can our observations on *E. coli* transcriptional network be generalised to other bacteria? To address this question, we have compiled orthologs of *E. coli* TFs across 216 non-redundant bacterial genomes (using the approach described in Ref. 67) and analysed their conservation. As a general observation, global regulators (and negatively autoregulated TFs) of *E. coli* tend to be more conserved than more specialised regulators (see Fig. 4). In spite of this observation, previous studies indicated that even the TFs found at the top of regulatory hierarchies vary across bacterial phyla.^67–69^ Intriguingly, orthologs of CsgD and FlhCD are found in less than 25 genomes, whereas orthologs for OmpR and FliA are found in more than 75 genomes. More precisely, FliA appears to be conserved in many organisms lacking FlhCD orthologs, although FlhCD is involved in the transcriptional regulation of FliA in *E. coli*. Tentatively, in these organisms, a different TF might regulate FliA. Alternatively, FliA alone might control the transcription of genes required in the final stages of motility and chemotaxis, or FliA might be directing the transcription of genes for unrelated functions in these bacteria. This set of regulatory components and the corresponding network structure are likely to be conserved in a relatively small group of bacteria (less than 25 bacteria closely related to *E. coli*). However, the general network organisation (long regulatory cascades *versus* multiple FFLs, and endogenous *versus* exogenous sensing) apparently constitute a common theme for the regulation of physiological and developmental processes in bacteria.

## Conclusions

Our structural analysis of the transcriptional cross-regulatory network in *E. coli* suggests that regulatory interactions between TFs are predominantly positive, while autoregulatory interactions are mostly negative. However, this general trend appears to be reversed in the case of most downstream TFs involved in the regulation of biofilm/chemotaxis modules.

We also note that there are striking topological differences between the subnetwork controlling metabolic activities, such as carbon metabolism, and that controlling developmental processes; the former encompasses many parallel short transcriptional cascades and multiple FFLs, each enabling the use of one alternative carbon source, while the latter involves long and intertwined regulatory cascades. These long transcriptional cascades typically include multiple autoactivated intermediate TFs, as well as regulatory circuits between TFs and sigma factors in the case of biofilm formation.

We further observe that TFs acting at the end of these regulatory cascades often belong to two-component systems. This topology suggests that cell homeostasy is maintained through multiple regulatory cascades with commonly autorepressed TFs, while the regulatory memory within the network is preserved by the sequential activation of TFs and by multi-element circuits at the core of the network. Downstream of the hierarchical network, two-component systems can memorise transient external signals through autoactivation loops, thus acting as molecular switches enabling the coexistence of alternative phenotypes.

As shown in a recent study, the *E. coli* cross-regulatory network appears to be robust enough to tolerate the rewiring between members high and low in the network hierarchy.^66^ This study also indicates that the allosteric signals are the mandatory input elements for network function. Thus, TFs present in a condition different from the natural one(s) would have limited activity due to the absence of their effector signals.

In this respect, a proper global understanding of the organisation of the *E. coli* transcriptional network (combining sigma and TFs) could contribute to the interpretation of network-rewiring experiments as well as foster more efficient design of synthetic regulatory circuits.

The general significance of the observed organisation of the *E. coli* transcriptional cross-regulatory network remains to be assessed. A more comprehensive picture of the network organisation in bacteria will progressively be drawn as additional regulatory elements such as small RNAs, anti-sigma factors and riboswitches are integrated.^70^ In addition, the combination of transcriptional and metabolic networks should provide important insights by linking effector metabolites and regulatory elements. Clearly, variations in regulatory network topology might be expected in the case of bacteria with asymmetric cell division (mostly α-proteobacteria), where the offspring asymmetric cells cause a transient genetic asymmetry that triggers different developmental processes, such as the formation of stalked and swarmer cells in *Caulabacter* or vegetative and spore-forming cells in *Bacillus*.^71–75^ Future comparisons between network topologies for different model systems should further enhance our understanding of regulatory network organisation and its conservation or variations among different bacterial phyla.

## Figures and Tables

**Table 1:** Distributions of positive, negative and dual (auto)regulatory interactions, mean path length^16^ and observed maximum out- and in-degrees for different sections of the *E. coli* transcriptional cross-regulatory network shown in Fig. 1

Network section	No. of TFs	Autoregulations	Positive autoregulations	Negative autoregulations	Dual autoregulation	Regulatory arcs^a^	Positive arcs	Negative arcs	Dual arcs	Average path length^b^	Maximum out-degree^c^	Maximum in-degree^c^
All	115	80 (70)	24 (30)	48 (60)	8 (10)	166	90 (54)	67 (40)	9 (6)	2.74	42	9
Carbon sources	24	19 (79)	5 (26)	9 (48)	5 (26)	27	20 (74)	6 (22)	1 (4)	1.53	20	2
Biofilm and motility^d^	32	18 (56)	9 (50)	9 (50)	0	52	22 (42)	27 (52)	3 (6)	3.12	8	9

Values in parentheses are percentages. Subnetworks for alternative carbon sources and for biofilm and chemotaxis development processes are defined in Fig. 2a. Note that while the carbon sources module has a relatively high maximum out-degree compared to the biofilm/motility module, the latter has a higher maximum in-degree, clearly suggesting that the motility TF, flhCD (with nine inputs), is directed by several TFs to control its regulation.

a Regulatory interactions from TFs to others TFs or towards sigma factors.

b Average path lengths in the (sub)network(s) were calculated with the ViSANT program.^16^

c Excluding sigmas.

d Only the TFs forming cascades ending on biofilm and chemotaxis modules were computed, the autoregulation of CRP was computed in the carbon sources module (Fig. 2a).

## Appendix A Supplementary data

Supplementary material Transcriptional cross-regulatory network analysed in this study, FFL motifs identified in the network, mRNA abundances and conservation of transcription factors are available in a supplementary excel file.
